# Genomic non-redundancy of the *mir-183/96/182* cluster and its requirement for hair cell maintenance

**DOI:** 10.1038/s41598-019-46593-y

**Published:** 2019-07-16

**Authors:** Joseph Fogerty, Ruben Stepanyan, Lauren T. Cianciolo, Benjamin P. Tooke, Brian D. Perkins

**Affiliations:** 1 Department of Ophthalmic Research, Cole Eye Institute, Cleveland Clinic, Cleveland, OH USA; 2 Department of Otolaryngology-Head and Neck Surgery, Case Western Reserve University School of Medicine and Ear, Nose & Throat Institute, University Hospitals Cleveland Medical Center, Cleveland, OH USA; 3 Department of Neurosciences, Case Western Reserve University School of Medicine, Cleveland, OH USA

**Keywords:** Hair cell, Genetics of the nervous system, Retina

## Abstract

microRNAs are important regulators of gene expression. In the retina, the *mir-183/96/182* cluster is of particular interest due to its robust expression and studies in which loss of the cluster caused photoreceptor degeneration. However, it is unclear which of the three miRNAs in the cluster are ultimately required in photoreceptors, whether each may have independent, contributory roles, or whether a single miRNA from the cluster compensates for the loss of another. These are important questions that will not only help us to understand the role of these particular miRNAs in the retina, but will deepen our understanding of how clustered microRNAs evolve and operate. To that end, we have developed a complete panel of single, double, and triple *mir-183/96/182* mutant zebrafish. While the retinas of all mutant animals were normal, the triple mutants exhibited acute hair cell degeneration which corresponded with impaired swimming and death at a young age. By measuring the penetrance of this phenotype in each mutant line, we determine which of the three miRNAs in the cluster are necessary and/or sufficient to ensure normal hair cell development and function.

## Introduction

miRNA primary transcripts are generated principally by RNA Polymerase II^1^ and form hairpin structures that are recognized and excised into pre-miRNAs by the “microprocessor,” a complex of proteins including the ribonuclease Drosha^2^ and the RNA binding protein Dgcr8^3^. This complex binds at the base of the hairpin and cleaves the duplex about 11 nucleotides from the branch point^4^. The pre-miRNA is then further processed by Dicer in the cytoplasm, which cleaves the unpaired end^5^. One of the resulting 18–22 nucleotide strands then associates with Argonaute to form the RISC complex, which facilitates mRNA targeting, typically within its 3′ untranslated region^6^.

The importance of miRNAs in the retina is vividly illustrated when components of the core miRNA biogenesis pathway are mutated. Targeted deletion of *Dicer* in mouse retinal progenitor cells leads to the organ’s developmental arrest and atrophy^7^. Similarly, restricting *Dicer* deletion to rod photoreceptors causes rapid photoreceptor degeneration^8^, and knocking out *Dgcr8* in cones (C-DGCR8-KO) has a similar effect in those cells^9^. RNA profiling of purified mouse cone photoreceptors was used to identify which miRNAs are expressed in those cells, and therefore give insight into which miRNAs might be most critical for cone cell survival. These experiments revealed that a single miRNA species, *miR-182*, accounts for 64% of all miRNA expression in that cell type^9^. A similar distribution was seen in human retina^10^. Impressively, combined re-expression of *miR-182* and closely-related *miR-183* was sufficient to rescue cone degeneration in C-DGCR8-KO mice^9^. Taken together, these studies indicate that not only is miRNA-dependent gene regulation of critical importance in the retina, but that several of these miRNAs make a compelling case for immediate investigation.

**Figure 1:**
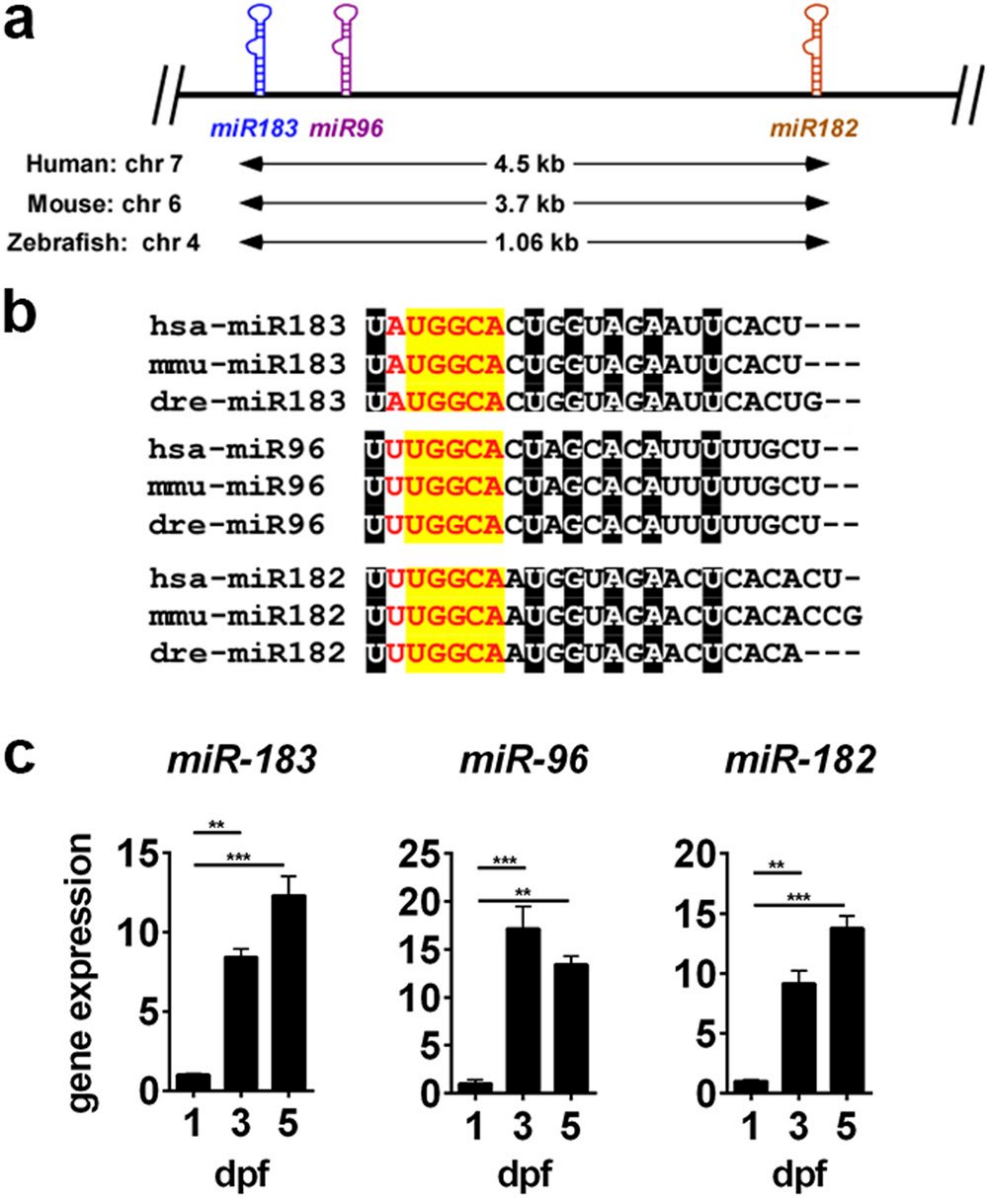
Primary sequence overlap, conservation, and expression of the *mir-183/96/182* cluster. (**a**) Schematic diagram illustrating genomic structure of the *mir-183/96/182* cluster. (**b**) Primary sequence of each miRNA in human, mouse, and zebrafish. The minimal seed sequence is displayed in red text. Nucleotides within the seed region with 100% conservation between component miRNAs and across species are highlighted in yellow. Nucleotides highlighted in black are similarly conserved, but are outside the seed sequence. (**c**) miRNA expression in zebrafish head tissue during early development was measured by qPCR. For all data points, n = 3. **p < 0.01, ***p < 0.001, ANOVA with Dunnet’s multiple comparisons test, relative to 1dpf.

*miR-182* is co-expressed along with *miR-183* and *miR-96* on a single primary transcript^11^ (Fig. 1a,b). This type of microRNA clustering is caused primarily by gene duplication events which can therefore lead to redundancies^12^, meaning that organisms are often unaffected by the loss of individual miRNAs from the cluster^13,14^. Even after sequence divergence occurs, clustered miRNAs can continue to evolve in parallel and maintain overlapping sets of target mRNAs due to the constraints of target availability and common selective pressures, thereby preserving those redundancies^15^. An example of this is the mouse *mir-17~92a* cluster, which contains 6 miRNAs that together regulate the expression of seven different subunits of voltage-gated potassium channels in spinal neurons. While overexpression of single *miR-17~92a* component miRNAs effectively downregulated target genes, the downregulation was enhanced when the entire cluster was overexpressed. Furthermore, downregulation of the entire cluster was more effective than single miRNAs at resolving allodynia^16^. These experiments indicate that although the seeds of these miRNAs have diverged and their targets have diversified, they cooperate by targeting different genes in a common pathway and thereby amplify a downstream effect. This cooperativity of multiple miRNAs to target multiple functionally related genes is considered a mechanism for the coordinated control of gene networks^17^.

Several studies have alluded to important roles that the *mir-183/96/182* cluster may play in the retina, as well as in other tissues. A gene trap that inactivated the entire cluster resulted in progressive photoreceptor degeneration and increased susceptibility to light damage^18^, and targeted deletion of *mir-183/96* had a similar effect^19^. A mouse expressing a “miRNA sponge,” in which *miR-183*, *miR-96*, and *miR-182* were all depleted by overexpression of a transgene encoding multiple miRNA binding sites (MBS) for each miRNA was unaffected under normal conditions but was also sensitized to high-intensity light^20^. Loss of only *miR-182* had a similar effect on light damage sensitivity, caused a decrease in ERG amplitude, and resulted in downregulation of several photoreceptor-specific genes^21,22^. This cluster is also highly expressed in hair cells^11,23^, and mutations in *mir-96* have been linked to hearing disorders in humans^24,25^, as well as in the *diminuendo* mouse^26^. This phenotype is also apparent in two mouse models in which the entire cluster was deleted^27,28^. In the zebrafish, hair cells in the ear permit perception of acceleration and rotation, as well as sound. In addition to the ear, bundles of hair cells that form on the head and trunk, called neuromasts, sense water movement along the body of the fish, and are important for schooling, predation, and predator avoidance^29,30^. Additional studies in zebrafish have implicated the *mir-183/96/182* cluster in the development of these organs^31^.

The relatively mild retinal phenotype of *mir-182*^−/−^ mice (compared to whole-cluster knockouts) suggests that those remaining miRNAs may partially compensate for the loss of *miR-182*. Indeed, of the hundreds of predicted *miR-182* targets, the study only identified five candidates that were significantly upregulated in the retina of *mir-182*^−/−^ mice that could be correlated to retinal disease^22^. Furthermore, computational analysis with TargetScan^32^ indicates that each of these five candidate genes harbor target sites for *miR-183* and/or *miR-96*, suggesting that loss of additional cluster components could exacerbate their misregulation and the accompanying retinal phenotype.

Despite the significant number of mutations generated and experimental manipulations performed on this cluster, no study has systematically evaluated the potential for either cooperativity or redundancy among the three component miRNAs, and synthesizing a unifying theory of the cluster’s function is difficult given the different models and methods employed. Given their high degree of similarity and their conservation throughout the evolutionary lineage, it is probable that these miRNAs work together in some way. Our objective in this study is therefore to understand which miRNAs in the *mir-183/96/182* cluster contribute to development and maintenance of sensory cells in the visual and auditory systems, and how they might cooperate with each other to do so.

The zebrafish is a well-established animal model for the study of human retinal disease^33–35^. Although zebrafish lack a fovea, its retina is very cone-rich relative to rodent models, making it a particularly useful model for the study of this cell type. Furthermore, the primary sequences of *miR-183*, *miR-96*, *and miR-182* are 100% conserved between zebrafish and mammals, suggesting that their functions are similarly conserved. The high fecundity of the zebrafish combined with the facility with which one can now generate targeted mutations make it well suited for experiments where multiple mutant lines need to be produced simultaneously. These qualities make the zebrafish the optimal model system in which to address our question.

## Results

### *miR*-183/96/182 expression in zebrafish larvae

*miR-183/96/182* is highly expressed in mammalian sensory cells, including those in the retina, inner ear, and dorsal root ganglia^11,18,36–40^. Within the mouse retina, they are expressed robustly in both photoreceptors and retinal interneurons^11^. To confirm the expression of these miRNAs in developing zebrafish larvae, we used qPCR assays designed to detect their mature products. In agreement with previous reports^31^, we found that each miRNA from the cluster is detectable in larvae as early as 1 day post-fertilization (dpf), and that their expression increases relative to a control snRNA through day 5 (Fig. 1c). We also observed persistent expression in adult zebrafish retina (not shown). We therefore concluded that the zebrafish is an appropriate system in which to proceed with our studies.

### *miR*-183, *miR*-96, and *miR*-182 are predicted to have a high degree of target overlap

To visualize potential target overlaps of these miRNAs in a broad context, we used CSmirTar (Condition-Specific miRNA Targets database)^41^ to identify human *miR-183/96/182* targets associated with the term “retinitis pigmentosa,” a blinding disease characterized by progressive photoreceptor degeneration that is similar to the phenotype observed in mutant mice. This yielded 125 unique genes, of which 70% had target sites for more than one miRNA in this cluster, and 44% had target sites for all three. As a comparison, we did an identical analysis using *miR-191*, *miR-26a*, and *miR-181b*, which are unclustered but are also highly expressed in photoreceptors^9^. These three miRNAs also had 125 unique targets linked to retinitis pigmentosa, but only 46% of them had target sites for more than one of the miRNAs, and only 13% had target sites for all three. (Supplementary Fig. S1). The substantially greater degree of overlap among *miR-183/96/182* targets is further evidence that they are likely to have functional overlap, which is a common feature of clustered miRNAs.

### Products of mutant *miR-183/96/182* alleles are expressed at extremely low levels and have impaired function

In mice, the loss of *miR-183* and *miR-96* in addition to *miR-182* produces a more severe retinal phenotype than loss of *miR-182* alone^18,22^, but the contribution of each miRNA remains unclear. To resolve this *in-vivo*, we generated a full panel of mutant zebrafish. Using a CRISPR/Cas9-based gene editing approach, we mutated each miRNA from the cluster both independently as well as in all possible combinations. This approach would allow us to systematically, in a single model organism, determine which of these three miRNAs are necessary and/or sufficient to ensure photoreceptor survival. We predicted that if *miR-183*, *miR-96*, and *miR-182* were completely redundant, only the triple mutant would exhibit any phenotype, as any single intact miRNA would compensate for the other two. Alternatively, if they act cooperatively, then single and double mutants would exhibit varying degrees of phenotypic severity or penetrance.

**Figure 2:**
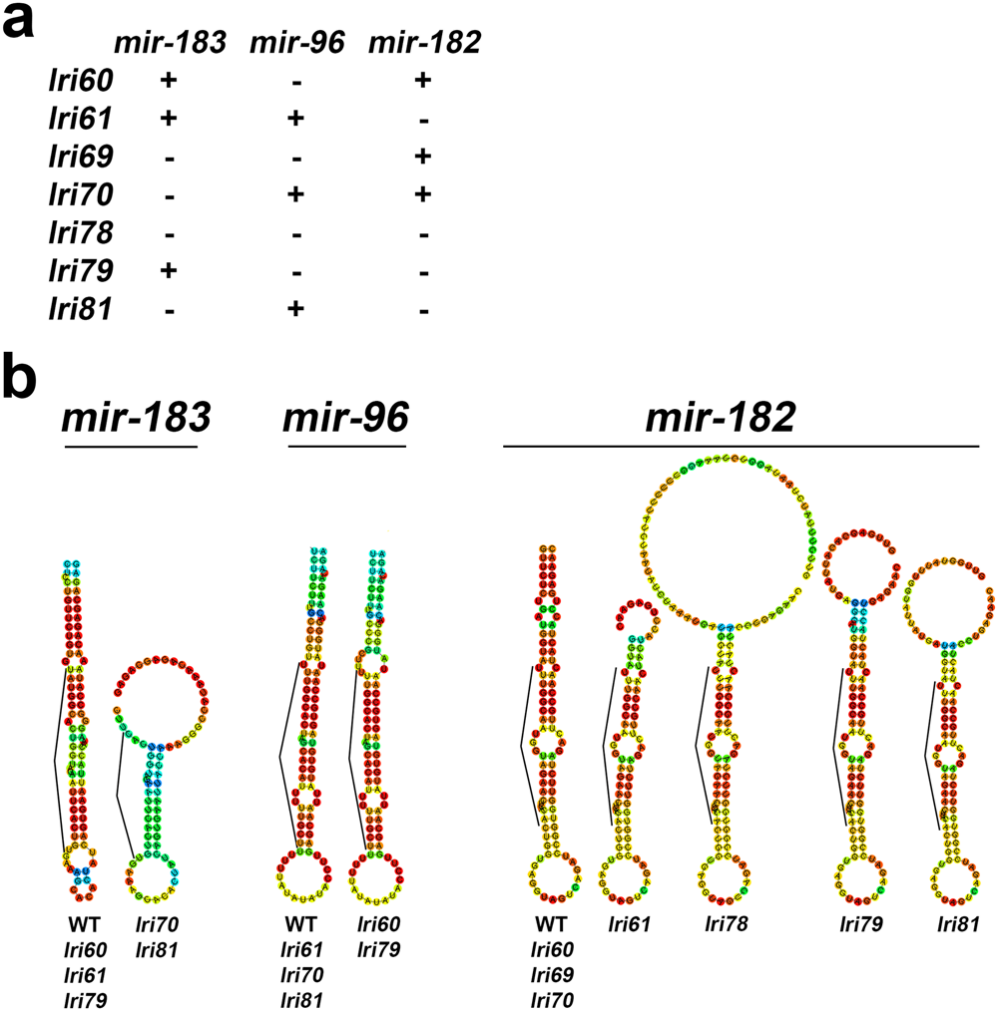
Summary of mutations generated in zebrafish *mir-183/96/182*. (**a**) Simplified table to illustrate which miRNAs are mutated in each allele. (**b**) Predicted secondary structures of wild-type and mutant miRNAs generated with CentroidFold. Warmer colors indicate higher base pairing probability. Listed below each structure are the alleles that harbor it. *lri69 and lri78* have no *mir-183* or *mir-96* because the region spanning both of these miRNAs was deleted. The bracketed region in each hairpin is the mature miRNA sequence, or, in the case of *mir-183* in *lri70* and *lri81*, its remnant.

In zebrafish, the conventional viewpoint has been that CRISPR/Cas9-induced double strand breaks are repaired by the non-homologous end joining pathway^42^, although recent evidence suggests that an alternative repair mechanism might be involved^43^. Either mechanism generates small insertions, deletions, and combinations thereof. The mutations we recovered varied in size from 2–15 base-pairs, plus one large deletion that eliminated both *mir-183* and *mir-96* (Supplementary Fig. S2, Fig. 2a). CRISPR/Cas9 targeting requires the presence of a 3-nucleotide PAM (protospacer adjacent motif) site in the target DNA sequence which is required for Cas9 binding, and therefore places restrictions on exactly where mutations can be generated^44,45^. This resulted in most of the mutations being slightly upstream of the mature miRNA sequence. However, we predicted that the mutations would nonetheless modify the hairpin structure in the primary transcript, and thus prevent normal miRNA expression by inhibiting or altering transcript processing by Drosha/Dgcr8. RNA structural models generated with CentroidFold^46^ supported this prediction (Fig. 2b). While the basic hairpin structure was preserved in each case, the stem was shortened such that the sequence of the mature miRNA was closer to the branch point. Importantly, the location of the branch point is the most critical factor in determining the place at which the Drosha/Dgcr8 complex cleaves the hairpin from the primary transcript^47^. Therefore, even if these mutant transcripts are indeed viable substrates for further processing, the pre-miRNAs generated from them would likely be truncated at both the 5′ and 3′ ends and would have dramatically altered targeting properties.

**Figure 3:**
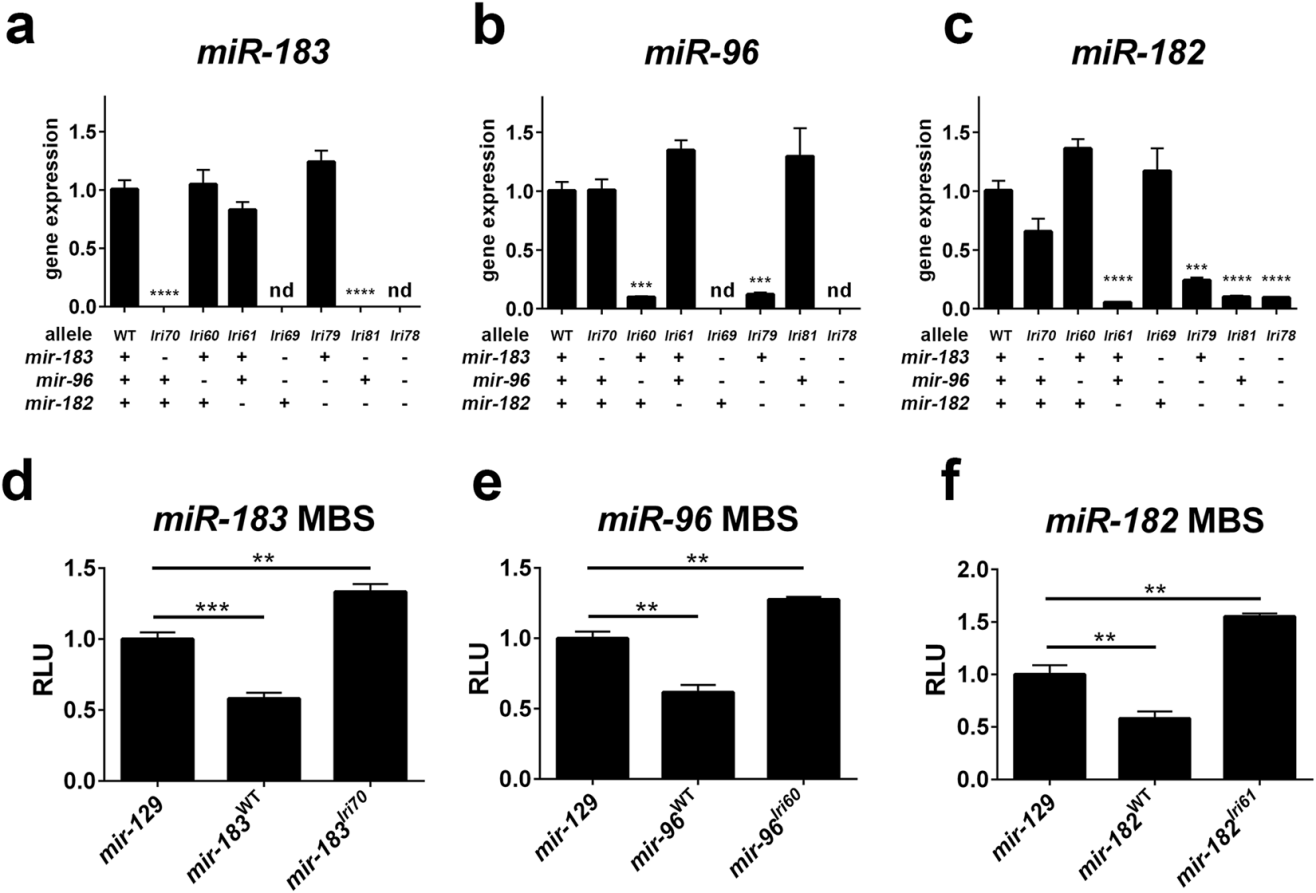
Expression and activity of mutant miRNAs. (**a**–**c**) Wild-type and mutant miRNAs were detected in adult zebrafish retina by qPCR. Mutant animals were all homozygotes. Asterisks indicate significantly decreased expression, relative to WT. nd = not detected. (**d**–**f**) miRNAs were cloned from adult mutant and wildtype genomic DNA and co-transfected with luciferase reporters encoding miRNA binding sites (MBS) for the indicated miRNAs. Decreased reporter signal indicates miRNA activity, relative to the *miR-129* control miRNA. RLU = relative light units. **p < 0.01, ***p < 0.001, ****p < 0.0001, ANOVA with Dunnet’s multiple comparisons test. For all data points, n = 3.

We tested the processing and function of these mutant miRNAs with two approaches. First, we tested the hypothesis that these mutations would decrease their expression by using qPCR to measure the relative abundance of the mature miRNAs in mutant zebrafish. We were unable to detect any *miR-183* or *miR-96* from the homozygous *mir-183/96*^*lri69*^ and *mir-183/96/182*^*lri78*^ mutants, each of which carry a large mutation that deletes both *mir-183* and *mir-96* in their entirety, confirming the specificity of the reaction. In all other cases, we detected low amounts of product, not exceeding 24 percent of that in wild-type animals (Fig. 3a–c). The degree to which these miRNAs were depleted in the mutants provided reasonable assurance that additional paralogs are not present in the zebrafish genome. It is notable that the *mir-96* mutation in the *mir-96*^*lri60*^ and *mir-96/182*^*lri79*^ alleles, which is a net insertion of only 2 nucleotides and decreased expression by 90 percent and 88 percent, respectively, was nearly as disruptive as the much larger *mir-182*^*lri61*^ mutation (11bp deletion). Because there was still detectable expression from most mutant alleles, it raised questions about whether they retained function. To address this, we used cultured HEK293 cells to test the functionality of a subset of these mutant miRNAs by co-transfecting them with a cognate reporter construct and measuring luciferase activity relative to a control miRNA. While wild-type miRNAs each decreased reporter activity by 38–42 percent relative to the control, mutant miRNAs did not (Fig. 3d–f). Taken together, these results suggest that although small amounts of miRNA-like molecules may still be processed from some of these mutant transcripts, their function is severely impaired. While we cannot rule out residual function of mutant miRNAs that had detectable expression, we suggest that they are at best severe hypomorphs.

### *mir-183/96/182* mutants have normal retinal structure and function

**Figure 4:**
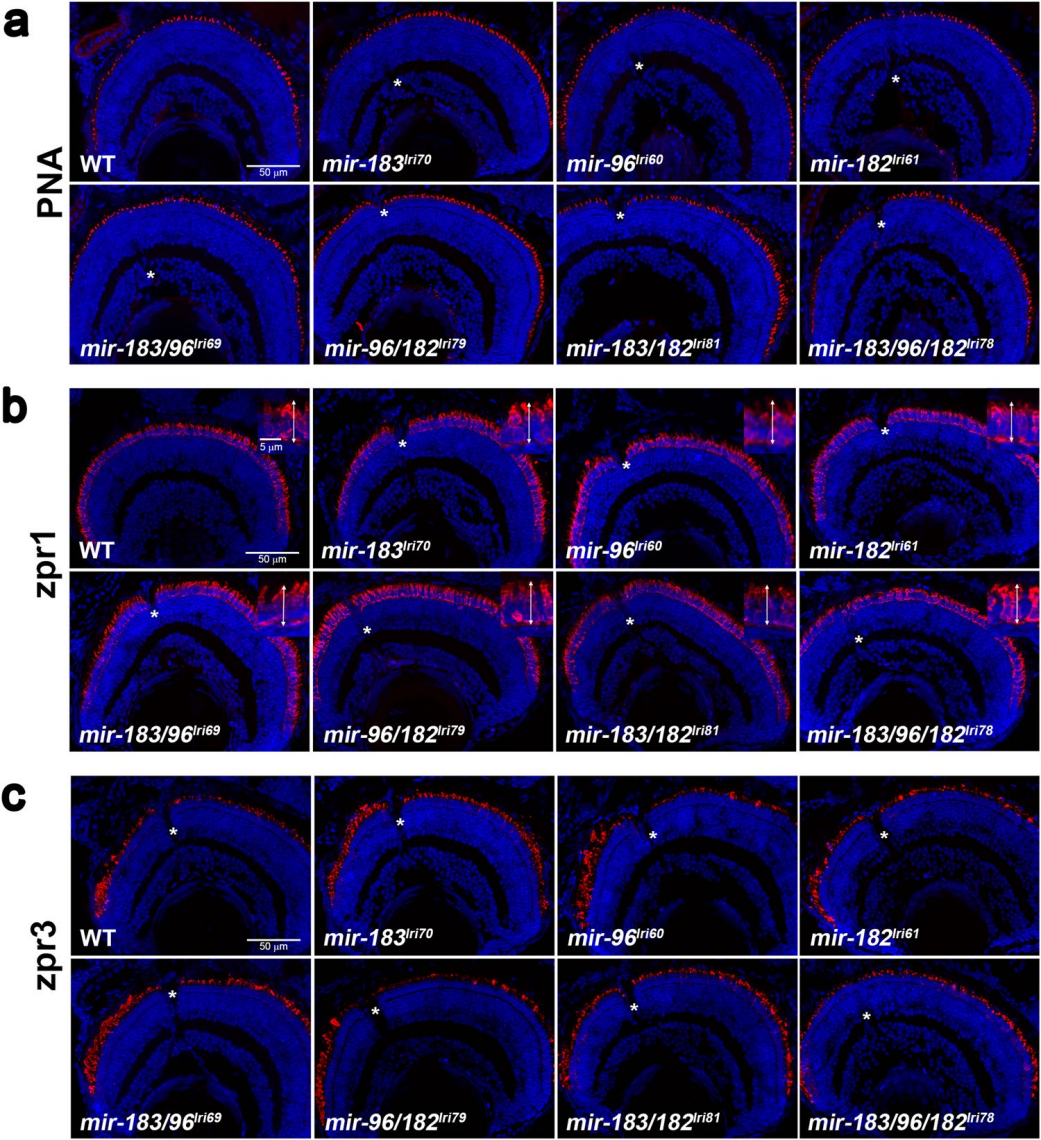
Survey of retinal anatomy in 5dpf mutant larvae. Transverse sections were collected near the optic nerve, which is visible in some images (asterisks), and stained with markers for different photoreceptor cell types. In all images, the ventral retina is to the left. (**a**) Peanut agglutinin lectin (PNA) labels the extracellular matrix surrounding cone outer segments. (**b**) zpr1 labels red/green cone inner segments. Inserts show higher magnification images, with double arrows indicating how inner segments were measured. (**c**) zpr3 labels rod outer segments, which are immature at this age and typically longer and more numerous in the ventral retina.

**Figure 5:**
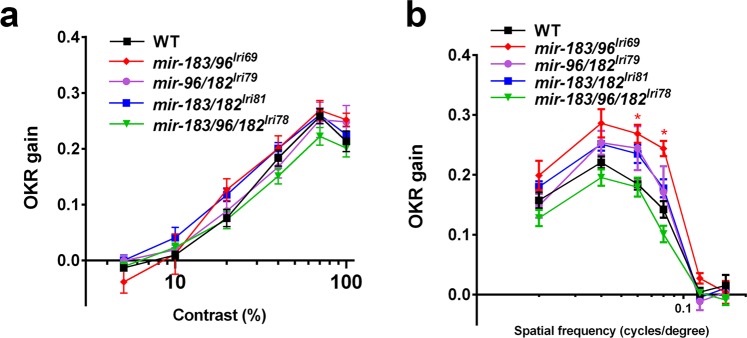
Mutant larvae have normal visual function. The optokinetic response (OKR) in 5 dpf larvae was measured while varying both (**a**) contrast and (**b**) spatial frequency of the stimulus. Asterisks in (**b**) indicate where a slightly improved OKR was measured in *mir-183/96*^*lri69*^ mutants. *p < 0.05, ANOVA with Dunnet’s multiple comparisons test.

Because we observed expression of these miRNAs in larval animals, and because their deletion has been shown to cause photoreceptor degeneration in other species^9,18,19,27^, we hypothesized that mutant zebrafish larvae would exhibit similar photoreceptor loss. We further predicted that if these miRNAs were functionally redundant, only the triple mutant would be affected, as the presence of any single component would compensate for the loss of the other two. We used peanut agglutinin lectin (PNA) to label cone outer segments, and the monoclonal antibodies zpr1 and zpr3 to label red/green double cone inner segments and rod outer segments, respectively, in 5 dpf fish. Although *miR-183* and *miR-182* were shown to be critical for proper cone outer segment development in mice^9^, we found no difference in the number of cone outer segments (Fig. 4a, Supplementary Fig. S3a) and only a modest shortening of *mir-183/96/182*^*lri78*^ cone inner segments (WT, 11.7 ± 0.4 μm; *lri78*, 10.3 ± 0.1 μm, Fig. 4b, Supplementary Fig. S3b). Rod outer segments, which are immature and have a non-uniform distribution in 5 dpf fish, were similarly unaffected (Fig. 4c). Loss of *miR-182* in mice caused a deficit in retina function but did not affect its structure^22^, so we also tested the *mir-183/96*^*lri69*^, *mir-96/182*^*lri79*^, and *mir-183/182*^*lri81*^ double mutants as well as the *mir-183/96/182*^*lri78*^ triple mutant for functional deficits in vision by evaluating the optokinetic response at 5 days post fertilization while varying the pattern contrast or spatial frequency. While the contrast response was normal for each mutant (Fig. 5a), we did see a slight, but significant, increase in the spatial frequency OKR gain of the *mir-183/96*^*lri69*^ mutant relative to wild-type larvae (Fig. 5b).

**Figure 6:**
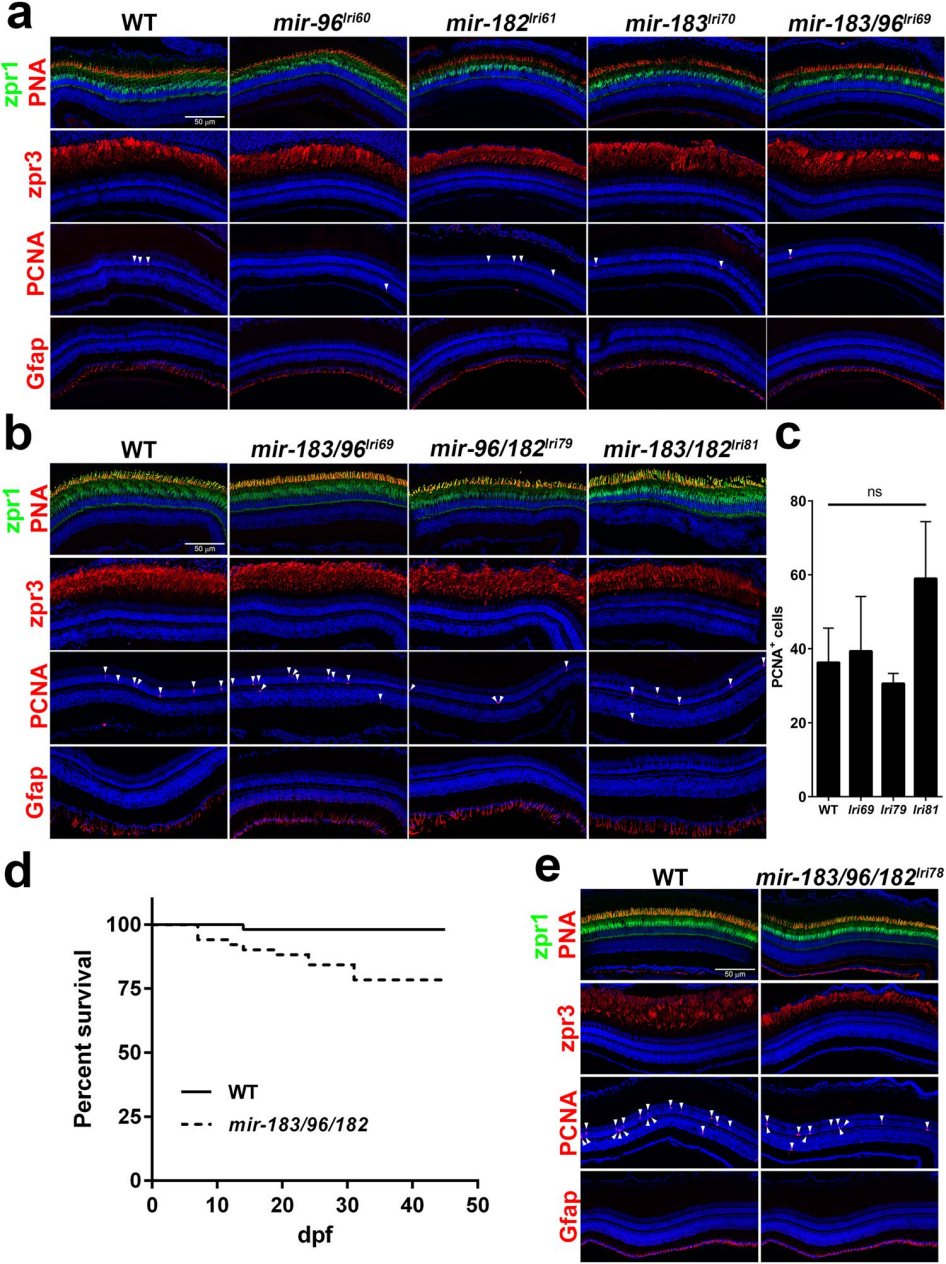
Adult mutants have normal retinal structure, but *mir-183/96/182*^*lri78*^ survival is depressed. (**a**,**b**) Survey of retinal anatomy in adult fish at (**a**) 12 m or (**b**) 6 m. Markers used here are equivalent to those used for imaging of larval fish, except that we also examined PCNA and Gfap staining to look for evidence of cell proliferation and gliosis, respectively. (**c**) Quantification of PCNA labeling in double mutants. PCNA + cells were counted across a 10μm thick retinal cross-section at the level of the optic nerve. Proliferating cells in the optic nerve head and in the ciliary marginal zone were excluded. ns = not significant (ANOVA). For all data points, n = 3. (**d**) Survival curve of fish from incrosses of WT and *mir-183/96/182*^*lri78*^ heterozygous parents. Each of the two cohorts began the study with a population of 51 fry. (**e**) Histological analysis of a surviving 15wk *mir-183/96/182*^*lri78*^ homozygote, showing normal retinal anatomy. Arrows in (**a**), (**b**), and (**e**) denote PCNA+ nuclei.

We then looked for age-related effects in older animals, as the existence of incomplete redundancies may cause a delay in disease progression. Each of the single mutants, as well as the *mir-183/96*^*lri69*^ double mutant, had normal numbers of cone outer segments (p = 0.074), normal cone inner segment length (p = 0.34), and normal rod outer segment length (p = 0.24) at 12 months (Fig. 6a, Supplementary Fig. S4a–c). The *mir-96/182*^*lri79*^ and *mir-183/182*^*lri81*^ double mutants also had a normal number of cone outer segments (p = 0.55), normal cone inner segment length (p = 0.07), and normal rod outer segment length (p = 0.23) at 6 months (Fig. 6b, Supplementary Fig. S4d–f). Teleosts, including zebrafish, possess the ability to regenerate retinal neurons^48^, which could potentially mask a slow degeneration. We looked for evidence of regeneration by immunostaining for PCNA, a marker of proliferating cells. PCNA staining was limited to 3–5 cells per section in all animals observed at 12 months, but was more extensive in all fish observed at 6 months, most likely due to more active retinal expansion in the younger animals. We quantified PCNA staining in the 6-month old *mir-183/96*^*lri69*^, *mir-96/182*^*lri79*^, and *mir-183/182*^*lri81*^ double mutants, but found that they were not significantly different from age-matched wild-type control animals (Fig. 6c). We also looked for changes in Müller cell activity by immunostaining for Gfap (Glial fibrillary acidic protein). These cells span the entire thickness of the retina and are important regulators of retinal homeostasis, and also play an integral role during the response to retinal injury in zebrafish^48^. This antibody typically stains the end feet of quiescent Müller cells along the vitreal surface of the retina, but that staining pattern will become more irregular in response to retinal damage^49^. Again, with each mutant we saw no notable morphological changes relative to control animals at any timepoint.

Of all of the mutants generated, only the *mir-183/96/182*^*lri78*^ triple mutant exhibited reduced viability, with many of these animals dying within several weeks post-fertilization. By raising fry from a *mir-183/96/182*^*lri78/+*^ incross at low density (3 fry/L) to minimize competition from wild-type and heterozygote siblings, we were able to generate a survival curve (Fig. 6d). While only a single animal was lost from a parallel cross of wild-type animals over the course of the 7-week study, animals from the mutant clutch, which was predicted to contain wild-type, heterozygous, and homozygous fish in a 1:2:1 ratio, gradually died until only 78% of the original population remained. This is in agreement with the expected Mendelian ratio if the homozygous mutation were lethal. The surviving fish were genotyped at the end of the study and while wild-type and heterozygous fish were found in the expected 1:2 ratio (n = 9 and 27, respectively; p = 1.125, χ^2^ goodness of fit test), we were surprised to also find four surviving homozygote animals. A pair of *mir-183/96/182*^*lri78*^ homozygotes that survived to 15 weeks were examined histologically with the same antibody panel that was used for the other mutants. Although the high mortality rate of the homozygotes precluded a more robust analysis, we again found nothing indicative of retinal degeneration beyond somewhat shorter rod and cone outer segments, which we attributed to the overall stunted growth of the mutants (Fig. 6e, Supplemental Video S1).

### Variable penetrance of hair cell degeneration in *mir-183/96/182* mutants is evidence of non-redundancy

**Figure 7:**
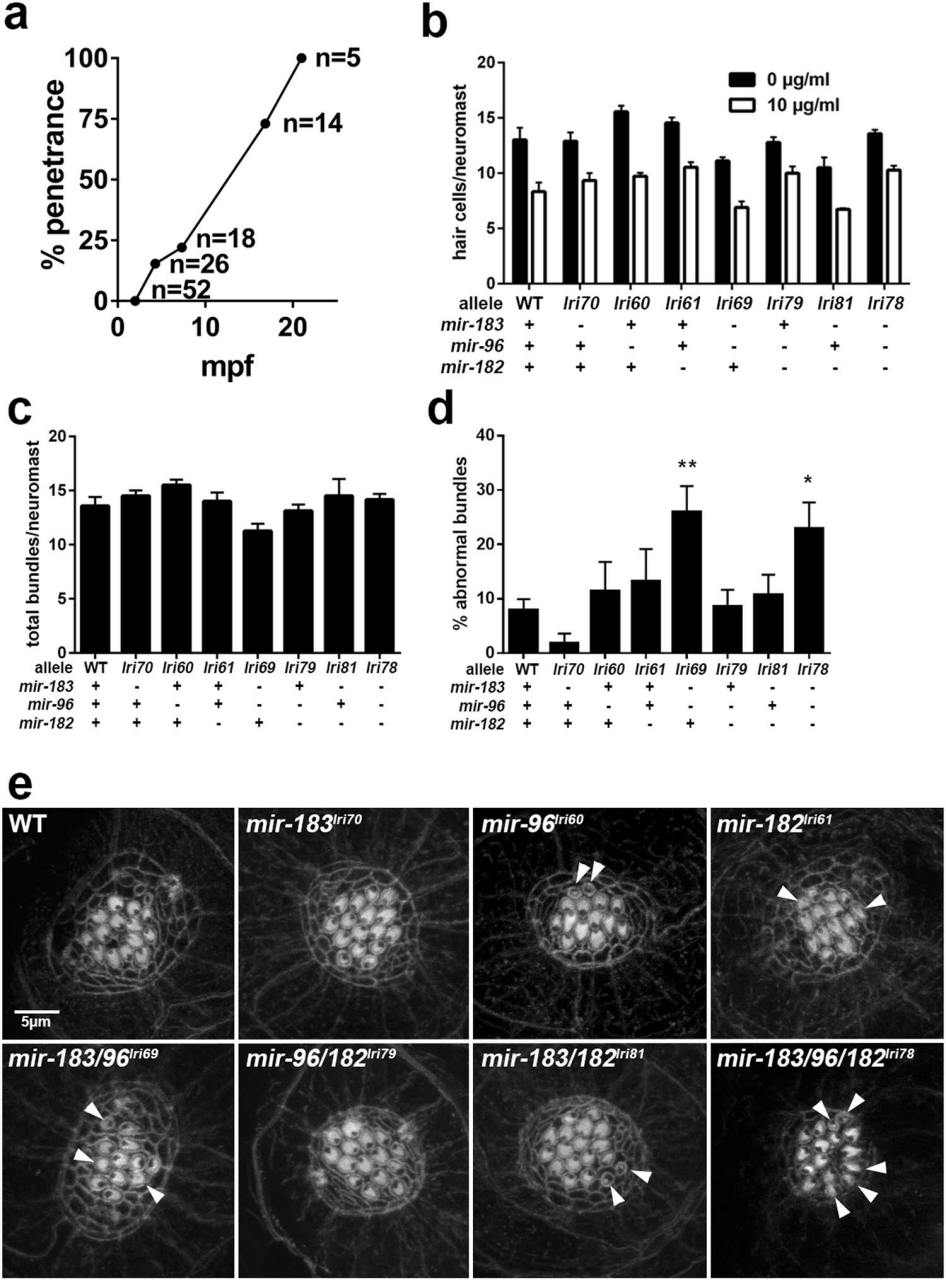
Abnormal hair cells in *mir-183/96*^*lri69*^ and *mir-183/96/182*^*lri78*^ mutants. (**a**) Penetrance of abnormal swimming phenotype in *mir-183/96*^*lri69*^ mutants in a cross-sectional survey of fish in our colony at the time of manuscript preparation. mpf = months post fertilization. (**b**) Acute toxicity of gentamicin on lateral line neuromast hair cells, as determined by HCS-1 immunolabeling. Differences among genotypes within each treatment group are not statistically significant compared to WT controls (ANOVA with Dunnet’s multiple comparisons test). Each data point is the average of 3–5 fish. (**c**) Counts of the total number of stereociliary bundles per neuromast. There are no statistically significant differences (ANOVA with Dunnet’s multiple comparisons test). (**d**) The fraction of neuromast hair cells with abnormal sterociliary bundles (*p < 0.05, **p < 0.01, ANOVA with Dunnet’s multiple comparisons test). For (d-e), sample sizes are: WT, 14; *lri70*, 4; *lri60*, 4; *lri61*, 4; *lri69*, 8; *lri79*, 8; *lri81*, 4; *lri78*, 13. (**e**) Representative images of neuromast stereociliary bundles, stained with phalloidin. Arrows indicate small or disorganized bundles that we scored as abnormal.

**Figure 8:**
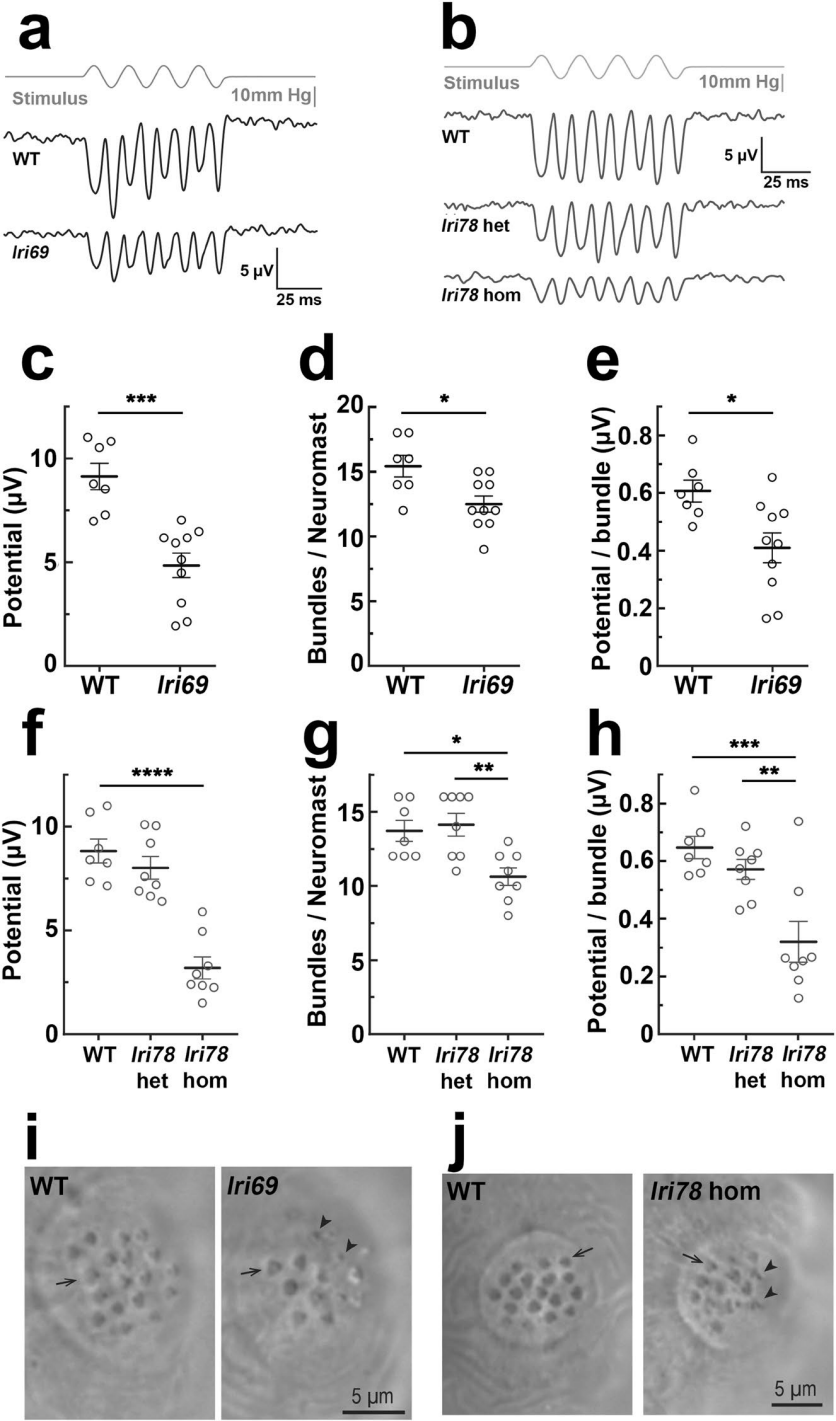
Microphonic potentials of *mir-183/96*^*lri69*^ and *mir-183/96/182*^*lri78*^ fish are reduced. (**a**,**b**) Representative microphonic potentials obtained from lateral line neuromasts of *mir-183/96*^*lri69*^ (**a**) and *mir-183/96/182*^*lri78*^ (**b**) fish. The top trace in each panel shows pressure applied to a stimulating puff pipette. Traces of microphonic potential recordings from neuromasts are shown below. (**c**) Summary of microphonic potentials from *mir-183/96*^*lri69*^ fish. Each point represents the average of maximal peak to peak amplitudes of responses produced from anterior and posterior deflections of the cupula. (**d**) Number of hair cells per neuromast in *mir-183/96*^*lri69*^ fish that were observable with brightfield imaging during recording. (**e**) Microphonic potentials from *mir-183/96*^*lri69*^ fish, normalized to the number of observable bundles in the neuromast. (**f**) Summary of microphonic potentials from *mir-183/96/182*^*lri78*^ fish. (**g**) Number of hair cells per neuromast in *mir-183/96/182*^*lri78*^ fish. (**h**) Microphonic potentials from *mir-183/96/182*^*lri78*^ fish, normalized to the number of observable bundles in the neuromast. (**I**,**j**) Representative neuromasts of *mir-183/96*^*lri69*^ (**i**) and *mir-183/96/182*^*lri78*^ (**j**) fish, imaged during recording. Arrows indicate normal stereocilia bundles, arrowheads indicate small and disorganized bundles. c-e, Student’s t-test; f-h, ANOVA with Tukey’s multiple comparisons test. *p < 0.05, **p < 0.01, ***p < 0.001, ****p < 0.0001.

We noted that the surviving *mir-183/96/182*^*lri78*^ homozygotes exhibited highly irregular swimming patterns. While their unaffected siblings typically swam in a 2-dimensional plane along the bottom of the tank, the mutants spun around and frequently changed direction (see Supplemental Video S1). Furthermore, adult *mir-183/96*^*lri69*^ mutants also exhibited this phenotype, although the age of onset was delayed by comparison and quite variable. We performed a cross-sectional survey of our *mir-183/96*^*lri69*^ mutant colony to identify animals with irregular swimming behavior and found that the penetrance of this phenotype within a clutch of animals was positively correlated with age (Fig. 7a, Supplemental Video S2). Given that this miRNA cluster is highly expressed in zebrafish hair cells and has been proposed to regulate their development^31^, and because these cells are critical for normal swimming behavior^50^, we suspected that the animals may be suffering from a hair cell disorder. To investigate this further, we characterized the structure and function of neuromast hair cells within the lateral line system in 5 dpf fish. These cells comprise the principal component of a mechanosensory organ that allows the fish to sense and respond to vibrations and changes in water velocity^51–54^. To assess hair cell morphology and their distribution within neuromasts, larvae were stained with phalloidin to label actin-based stereocilia, and the monoclonal antibody HCS-1, which recognizes an epitope on the hair cell plasma membrane^55^. We also measured their sensitivity to gentamicin, an ototoxic aminoglycoside antibiotic that causes neuromast hair cell death in a dose-dependent manner^56^, as some miRNA mutant phenotypes only become evident when the animals are challenged with additional stressors^57,58^. All mutants had neuromasts with the appropriate complement of hair cells, and none were excessively sensitive to gentamicin when we used a dose that was sufficient to produce a moderate amount of hair cell death in wild-type fish (Fig. 7b). This finding is in contrast with a previous report that found that knockdown of these miRNAs with morpholinos reduced the number of posterior neuromasts as well as the number of HCS-1^+^ hair cells in the neuromasts that remained^31^, but we suggest that our genetic ablation approach is a more reliable method for studying gene function, as it is difficult to control for off-target effects of morpholinos^59^. We then looked more directly at neuromast hair cell stereocilia with phalloidin staining, and found that all fish had the expected number of bundles per neuromast (ANOVA, p = 0.28, Fig. 7c). However, while wildtype animals had stereocilia that formed exquisitely organized semicircular bundles, those of the *mir-183/96*^*lri69*^ and *mir-183/96/182*^*lri78*^ mutants had an abnormally large proportion of hair cells whose stereocilia were either reduced to a single spot or lacked that characteristic arrangement, and are possibly a combination of immature, degenerating, and incompletely polarized cells. (WT, 7.9 ± 2.0% vs *mir-183/96*^*lri69*^, 25.9 ± 4.9% and *mir-183/96/182*^*lri78*^, 25.4 ± 3.2%, Fig. 7d,e). Not surprisingly, these were the same mutants that exhibited circular swimming behavior that is consistent with a hair cell disorder. To determine if these structural changes resulted in compromised hair cell function, we recorded microphonic potentials from lateral line neuromasts in response to fluid jet stimulation of wild type, *mir-183/96*^*lri69*^, and *mir-183/96/182*^*lri78*^ zebrafish larvae at 5–6 dpf. This potential is an extracellular voltage change resulting from current flow through activated mechanotransduction channels of hair cells. Deflection of kinocilia by fluid jets evoked a potential at double the frequency of stimulus deflections (Fig. 8a,b), alternately exciting hair cells of opposite orientation. Recordings from *mir-183/96/182*^*lri78*^ heterozygotes showed stimulus-evoked microphonic potentials equal in average to wild-type (8.01 ± 0.51 and 8.83 ± 0.53 µV, respectively), while homozygous *mir-183/96/182*^*lri78*^ larvae neuromasts had average potential of 3.19 ± 0.49 µV (Fig. 8f). The number of stereocilia bundles in the neuromasts of homozygous *mir-183/96*^*lri69*^ and *mir-183/96/182*^*lri78*^ fish that were observable with brightfield imaging was moderately reduced in comparison to wild type or heterozygous larvae (Fig. 8d,g,i,j), consistent with our observation of significantly more structurally abnormal stereocilia in these animals. Further analysis shows that the microphonic potential per bundle is significantly reduced in neuromasts of homozygous *mir-183/96*^*lri69*^ (Fig. 8e, 0.410 ± 0.052 µV vs. 0.607 ± 0.038 µV) and *mir-183/96/182*^*lri78*^ fish (Fig. 8h, 0.321 ± 0.066 µV vs. 0.648 ± 0.036 µV). Therefore, the reduction of microphonic potentials and the corresponding behavioral phenotype in these two mutants is likely due to nonfunctional or degenerating mechanosensitive hair cell bundles in lateral line neuromasts.

## Discussion

There is considerable evidence that *mir-183/96/182* is critical for photoreceptor maintenance in the mouse. Thus far, there have been two reports of independently generated lines in which all three of these miRNAs have been eliminated. Both lines exhibit some form of retinal pathology^18,27^. It is surprising that the zebrafish mutants we generated did not, but we can speculate about why this is the case. Importantly, we cannot rule out that the mutant miRNAs described here retain some residual function, as our reporter assays may lack the sensitivity to detect that. It is unlikely that additional paralogs are compensating for their loss, as BLAST searches for any of the individual miRNAs yielded no candidates with matching seeds that would be predicted to form hairpins. Furthermore, our own analysis of miRNA expression in mutant zebrafish makes us confident that we targeted the sole copies of these miRNAs in the genome, and the hair cell phenotype in *mir-183/96*^*lri69*^ and *mir-183/96/182*^*lri78*^ mutants corroborates that as well. It is possible that the early lethality of the *mir-183/96/182*^*lri78*^ mutant precluded our ability to observe any late-onset retinal phenotypes in those animals. This could be resolved by re-expressing one or more of the miRNAs in hair cells so as to permit longer survival. Minor or late-onset retinal phenotypes, such as that in the *mir-183/96/182* “sponge” mouse^20^, may require magnification or acceleration by stressors such as high intensity light before becoming apparent^57,58^. Finally, it is also possible that teleosts and tetrapods have evolved alternative mechanisms of photoreceptor maintenance since their divergence from a common ancestor about 450 million years ago, which may not share reliance on this cluster. In this case, a thorough investigation of *miR-183/96/182* targets in zebrafish as well as mammals is warranted.

In the zebrafish genome, approximately 50% of all miRNAs are positioned within 10 kB of another miRNA^15^, a phenomenon that is prevalent in all other species as well, although to varying degrees. Multiple theories have been put forth to explain the emergence and evolution of clustered miRNAs, including *de novo* birth and local and *trans-*duplication^12^. Since individual miRNAs within a cluster are so tightly linked, there is little chance of recombination between them, which helps to preserve the cluster’s structure and prevents components from being lost^60^. Furthermore, any mutations that randomly arise in any cluster component that interfere with its expression will be selected against, as there is always pressure to preserve at least one functional copy of the ancestral miRNA. This provides a safe harbor in which young miRNAs can evolve, and once an advantageous function emerges, positive selective pressure further promotes its retention^60^. Because clustered miRNAs are typically co-expressed, they are exposed to the same pool of target mRNAs, and it is therefore likely that they acquire not only overlapping sets of targets, but also that their discrete target sets include genes with related functions. Indeed, four miRNAs with unique seeds from the *mir-17~92a* cluster, when transfected into cultured cells, each caused similar changes to the transcriptional profile^15^. Additionally, genes affected by individual miRNAs from the cluster could be grouped into discrete functional units^15^. The same study found that genes targeted by multiple miRNAs within a cluster are expressed at lower levels than genes targeted by only one. This lends strength to the theory that clustered miRNAs cooperate to regulate biological pathways in two ways: by co-targeting single mRNAs and by targeting separate genes that participate in a common mechanism, and is in agreement with our own observations in which hair cell phenotype penetrance in zebrafish was correlated with loss of additional components of the *mir-183/96/182* cluster. Cooperative activity of these miRNAs likely includes actions on overlapping as well as discrete target sets, although our study cannot distinguish between the two.

In conclusion, we return to our original objective: to define the minimal requirement of these miRNAs for normal retinal development and function. The absence of a retinal phenotype in any of the mutants indicates that, at least in zebrafish, these miRNAs are collectively dispensable for that tissue. Our study is consistent with previous reports describing their role in hair cells^24–26,31,61,62^, and expands on those findings by shedding light on their relative contribution to the support of those cells. Because neither the *mir-183*^*lri70*^, *mir-96*^*lri60*^, nor *mir-182*^*lri61*^ mutants had compromised hair cells, we can also conclude that there is no absolute requirement for any one of the three miRNAs. Furthermore, both *miR-183* and *miR-96* are each sufficient to maintain cluster function, as neither the *mir-96/182*^*lri79*^ nor the *mir-183/182*^*lri81*^ mutants suffered any deficiencies either. The minor phenotype in the *mir-183/96*^*lri69*^ mutant, however, indicates that *miR-182* is the least capable of maintaining the function of the entire cluster by itself. This suggests that one or more targets regulated by *miR-183* and *miR-96* but not *miR-182* are crucial for normal hair cell function. Again using CSmirTar, we filtered predicted *miR-183/96/182* target genes with the disease term “sensorineural hearing loss,” and found two genes that meet these criteria, *FGFR1*, which may regulate patterning of the developing cochlear duct^63^, and *MYO6*, which is expressed in mature hair cells and is critical for the integrity of stereocilia^64^. Further insight into the biology of these miRNAs will be gained by comprehensively examining their potential targets and identifying additional common genes and pathways that they may co-regulate. This set of mutant zebrafish will be a useful tool to empirically test new hypotheses that arise from that analysis. Furthermore, to our knowledge we have generated the first complete set of mutant animals for any vertebrate miRNA cluster. They will therefore be a valuable resource for the study of clustered microRNAs in general, and will broaden our understanding of how they evolve and interact.

## Methods

### Zebrafish husbandry

Adult zebrafish were maintained and raised on an Aquatic Habitats recirculating water system (Pentair; Apopka, FL) in a 14:10-hour light-dark cycle. The Cleveland Clinic Institutional Animal Care and Use Committee (IACUC) approved all experimental procedures and ensured they were performed in accordance with relevant regulations.

### Real-time quantitative PCR

RNA from 10 pooled larval heads or single isolated adult retinas was prepared with Trizol (ThermoFisher, Waltham, MA). Three biological replicates were used for all experiments. cDNA preparation and PCR amplification was done on a CFX96 thermal cycler (Bio-Rad, Hercules, CA) using validated Taqman miRNA assays (ThermoFisher) for *miR-183* (Assay ID 000484), *miR-96* (000186), and *miR-182* (000597). *U6 snRNA* (001973) was used as a reference gene. Fold-change values were calculated using the ΔΔCt method.

### Generation of zebrafish mutants with CRISPR/Cas9

CRISPR targeting reagents were designed following established protocols^44^. Briefly, CRISPR target sequences were designed with ZiFiT and complementary oligos encoding the sequence were cloned into the BsaI site of pDR-274. Constructs were linearized with BsaI and gRNAs were synthesized using the MegaShortScript T7 kit (ThermoFisher). gRNA was purified by phenol:chloroform extraction and ammonium acetate/ethanol precipitation. We diluted the gRNA to 400 ng/ul and mixed it 1:1 with 20 μM recombinant Cas9 protein (New England Biolabs, Ipswich, MA), allowed a 5 minute incubation at room temperature to form complexes, and then injected 1nL into freshly fertilized zebrafish embryos^65^. To generate single mutants, gRNAs targeting each miRNA were injected into wildtype embryos. The *mir-183/96*^*lri69*^ double mutant was a large deletion spanning both loci that was recovered from a wildtype embryo in which *mir-183* was targeted. The remaining double and triple mutants were generated in a second round of mutagenesis by targeting *mir-182* in embryos from the previously established mutant lines. F1 fish carrying novel alleles were identified first by high-resolution melt analysis using PrecisionMelt master mix (Bio-Rad), followed by sequencing. Once mutant lines were established, genotyping was carried out by electrophoresis with 3% agarose gels, which gave sufficient resolution to distinguish all mutant alleles except *mir-96*^*lri60*^. This small indel was genotyped by high-resolution melt analysis. Sequences for all oligos are included in Supplementary Table S1. With the exception of the *mir-183/96/182*^*lri78*^ mutant, which was maintained with heterozygous crosses, all lines were maintained as homozygotes. Control animals for all experiments were age-matched WT animals from the same background strain as the mutants.

### Luciferase reporter constructs and assays

Reporter constructs were generated by first inserting a SanDI site into p3E-polyA via site directed mutagenesis (GeneArt, ThermoFisher), generating p3E-SanDI. Following the protocol described by Kluiver and colleagues^66^, oligonucleotides encoding a pair of miRNA binding sites (MBS) were inserted into the SanDI site of p3E-SanDI. All constructs used here contained a single insertion encoding two MBS. MBS specificity was confirmed by using the PITA algorithm^67^ to match each MBS to a database of 255 zebrafish mature miRNAs annotated in miRBase release 20^68^. We then recombined the MBS vectors with p5E-CMV/Sp6, pENTR-luc, and pDEST-Tol2pA2 using the Gateway three-fragment vector construction kit (ThermoFisher) to generate the completed reporters. To generate miRNA expression constructs, miRNAs were cloned from wildtype and mutant zebrafish genomic DNA and ligated into the EagI/Bsu36I site of pEF-GFP. All luciferase assays were conducted in HEK-293 cells in 24-well plates. Cells were grown to confluence and then transferred to serum-free medium. Each well was transfected with 50 ng pRL-SV40, 25 ng reporter, and 65 ng miRNA, in triplicate, using Lipofectamine 2000 (ThermoFisher). We found that reporter output was proportional to the amount of reporter DNA transfected up to a saturation point between 50 and 100 ng. Beyond that threshold, miRNA efficacy was compromised, even when using excess miRNA template. By using only 25 ng reporter plasmid, we ensured that the assays were conducted within the linear range to maintain miRNA sensitivity. 24 hours post-transfection, cells were lysed and assayed for normalized luciferase expression using the dual-luciferase reporter assay system (Promega, Madison, WI) with a Victor X2 plate reader (Perkin Elmer, Waltham, MA). pENTR-LUC (Addgene plasmid # 17473) was a gift from Eric Campeau & Paul Kaufman^69^. pEF-GFP (Addgene plasmid # 11154) was a gift from Connie Cepko^70^. pRL-SV40P (Addgene plasmid # 27163) was a gift from Ron Prywes^71^. p3E-polyA, p5E-CMV/Sp6, and pDestTol2pA-2 are components of the zebrafish Tol2kit^72^. Sequences for all oligos are included in Supplementary Table S1.

### Immunohistochemistry

Zebrafish larvae were fixed for 2 hours at 4 °C in PBS containing 4% paraformaldehyde, equilibrated in PBS + 30% sucrose, embedded in Tissue Freezing Medium (Electron Microscopy Sciences, Hatfield, PA), and sectioned at 10 μm thickness. Adult eyes were likewise fixed 2 hours in PBS + 4% paraformaldehyde, but were more gradually equilibrated by first incubating 3 hours in 5% sucrose before transferring them to 30% sucrose overnight. Adult eyes were then washed overnight in a 1:1 mixture of 30% sucrose:TFM before embedding. Transverse sections including or adjacent to the optic nerve were cut at 10 μm thickness and blocked 1 hour in PBS containing 2% BSA, 5% goat serum, 0.1% Tween-20, and 0.1% DMSO. Primary antibody incubation was carried out overnight at 4 °C in blocking buffer. Antibodies used are as follows: zpr1 (1:100, ZIRC, Eugene, OR), zpr3 (1:100, ZIRC), zrf-1 (1:100, ZIRC), PCNA (1:100, Sigma, St. Louis, MO, clone PC-10). PNA lectin conjugated to Alexa-568 (ThermoFisher) was used at 1:100 in blocking buffer. All secondary antibodies were from ThermoFisher and were used at 1:500 in blocking buffer with overnight incubations at 4 °C. Sections were counterstained with DAPI and imaged on a Zeiss Imager Z.2 with Apotome attachment, and post-processed in ImageJ. For cone inner segment analysis, we measured the length of five zpr1^+^ cells at regular intervals across the retina. Rod outer segment length was measured similarly with zpr3 imaging. Cone outer segments and PCNA^+^ nuclei were counted across the entire retina. All experiments used between 3 and 6 animals per genotype.

### Optokinetic response (OKR)

OKR measurements were recorded with a Visiotracker (TSE Systems, Bad Homburg, Germany) as previously described^73^. Each data point is the mean of measurements from at least 6 different animals.

### Gentamicin treatment

Live 5 dpf larvae were immersed in fish water containing 10 µg/ml gentamicin (ThermoFisher) for 10 minutes, which we found was sufficient to kill approximately 40% of the hair cells in wild-type animals. They were then rapidly washed 3 times in fish water, immediately fixed in 4% paraformaldehyde and stained and imaged as described below.

### Whole mount immunostaining

Zebrafish larvae were raised to 5 dpf, fixed overnight in PBS + 4% paraformaldehyde at 4 °C, and blocked 2 hours in PBS + 5% goat serum + 1% Tween-20. To label stereocilia, samples were stained overnight at 4 °C with Alexa488-phalloidin (ThermoFisher) diluted 1:50 in blocking buffer with gentle agitation followed by five 1-hour washes in blocking buffer. Stereocilia were imaged on a Leica SP8 confocal microscope (Leica Microsystems, Buffalo Grove, IL). To image hair cell somas, similarly prepared larvae were labeled with HCS-1 (DSHB, Iowa City, IA, 1:100) overnight in blocking buffer, washed, and then incubated overnight in anti-mouse-Alexa568 secondary antibody (ThermoFisher, 1:500). Samples were imaged on a Zeiss Axio Imager.Z2 with Apotome.2 attachment for structured illumination. Post-processing was done with ImageJ and Adobe Photoshop. Counts of HCS-1-positive hair cells were averaged from six neuromasts per fish, including three posterior neuromasts and three supra- or sub-orbital neuromasts. All scoring and quantification was performed by two independent individuals on images with identifying information removed.

### Measurement of neuromast microphonic potentials

Recordings of microphonic potentials^50,74^ were conducted as previously described^75^. Zebrafish larvae were anesthetized using ethyl 3-aminobenzoate methanesulfonic acid (Sigma-Aldrich) dissolved in a bath solution (120 mM NaCl, 2 mM KCl, 10 mM HEPES, 2 mM CaCl_2_, 0.7 mM NaH_2_PO_4_ and adjusted to pH 7.3). The larvae were secured in a recording chamber using strands of dental floss tie downs and placed under the microscope for observation. Zebrafish were visualized on an upright microscope (BX51WI; Olympus) equipped with 4 × 0.13 NA and 100 × 1.0 NA objectives. We monitored heart rate and circulation of blood cells to ensure the viability of larvae during recording. To view and capture images we used a Grasshopper3 CMOS camera (FLIR) and manufacturer provided software. We recorded microphonic potentials using a PC-505B amplifier (Warner Instruments), SIM983 amplifier (set at 20× , Stanford Research), and a PCI-6221 digitizer (National Instruments). Potentials were recorded from posterior neuromasts of larvae ranging from 5 to 6 dpf at room temperature (~22 °C). We used a borosilicate glass pipette (World Precision Instruments) with a resistance of 3–6 MΩ when filled with bath solution. This recording pipette was placed near the apical edges of the neuromasts. Kinocilia tufts were deflected with a fluid jet delivered via an additional glass pipette with a diameter of approximately 10 µm, which was controlled by jClamp software (SciSoft) and driven by HSPC-1 (ALA Scientific Instruments). A fluid jet pipette was placed approximately 80 µm away from the neuromast and used to deliver sinusoidal stimuli of 50 Hz generated by jClamp. Placement of fluid jet and recording pipettes were controlled by a set of micromanipulators (MPC-325; Sutter Instrument). Microphonic potentials were recorded with a jClamp software in current-clamp mode, and low-pass filtered at 200 Hz. All records represent an average of at least 500 trials.

## Supplementary information

Supplementary Video S1

Supplementary Video S2

Supplementary Information

## Data Availability

All datasets, plasmids and animal models generated in this study are available upon request from the authors.
