# Supplementary material for: Genomic non-redundancy of the mir-183/96/182 cluster and its requirement for hair cell maintenance
Source: Sci Rep. 2019 Jul 16;9:10302. doi: 10.1038/s41598-019-46593-y (PMC6635406; doi:10.1038/s41598-019-46593-y)
Supplement: Supplementary file 3 — Supplementary Information [file 41598_2019_46593_MOESM3_ESM.pdf]

Supplementary Information

Genomic non-redundancy of the *miR-183/96/182* cluster and its requirement for hair cell maintenance

Joseph Fogerty[1], Ruben Stepanyan[2,3], Lauren T. Cianciolo[1], Benjamin P. Tooke[1], and Brian D. Perkins[1]

[1]Department of Ophthalmic Research, Cole Eye Institute, Cleveland Clinic, Cleveland, OH

[2]Department of Otolaryngology-Head and Neck Surgery, Case Western Reserve University School of Medicine and Ear, Nose & Throat Institute, University Hospitals Cleveland Medical Center, Cleveland, OH

[3]Department of Neurosciences, Case Western Reserve University School of Medicine, Cleveland, OH

a

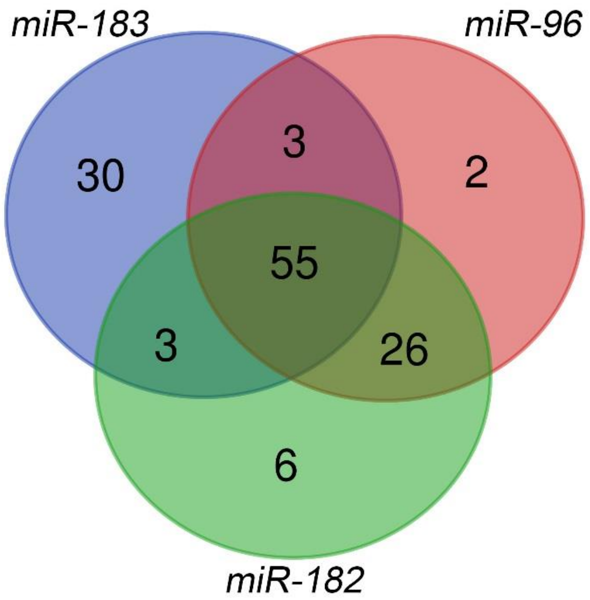

miRNA(s)	total	common targets
182 183 96	55	PITPNM3 CDHR1 CNOT3 BBS1 EYS KIDINS220 ARHGEF7 TIMP3 CRB1 IDH3B RP1L1 CCDC66 MYO7A GUCA1A SOS1 SMC1A NPTX2 ADAM9 NPHP3 AIPL1 BBS2 SPATA7 C2orf71 SEC14L1 SLC6A6 RIMS1 PROM1 USH2A AGTPBP1 FGF5 ALDH3A2 SOS2 STC1 ATXN3 ERG NPHP4 SEMA6A WDR19 ATXN7 TIMP2 TOPORS GUCA1B HK1 MAK PRPF3 MAPRE2 ALPK3 ARHGEF2 RASGRF1 ATXN2 BBS9 PCDH15 TUB GCNT2 CHM
183 96	3	IDH2 COG4 RAB8A
182 183	3	GPR98 CC2D2A EFTUD2
182 96	26	SNRNP200 DYNLT3 BEST1 COPS5 CANX RCC1 DHX38 PDE6A RCVRN PRPH2 STK19 POC1B DNAH8 LCA5 UNC119 CNTF RDH5 EDNRA LRAT PMM2 IMPG2 SYNJ1 RBP1 CERKL MT2A FAM161A
183	30	FAM46A MVK MERTK CP CEP290 ARPP21 TTC8 BBS4 OTC PRCD ABCG2 CNGB1 NUDT19 TTPA CDH23 RDH12 GUCY2D CNNM4 PRPF6 ALPPL2 SNRPB NT5C2 CNGA1 AHI1 SLU7 MAPRE3 RP2 KIZ BRS3 RAB28
96	2	PRPF31 SMC3
182	6	ALDH7A1 RP1 NRG4 PRPH PHEX APOB

b

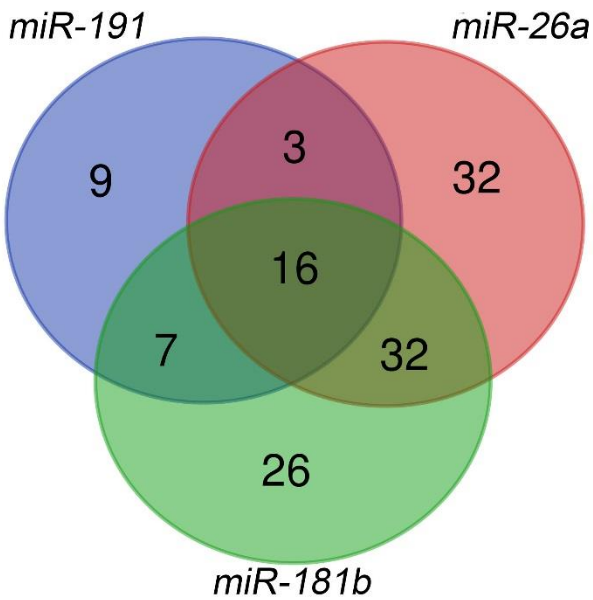

miRNA(s)	total	common targets
181b 191 26a	16	SNRNP200 CANX TIMP3 RPE65 SOS1 SMC1A ADAM9 BBS2 DLL1 C2orf71 SLC6A6 RIMS1 ATXN7 MAPRE2 BBS9 GCNT2
191 26a	3	ELOVL4 CRB2 RASGRF1
181b 191	7	PRPH2 CCDC66 CNGB1 CLN3 RDH12 ERG SLU7
181b 26a	32	IFNG EYS ARHGEF7 CP ARPP21 CRB1 RPGR BBS4 DNAH8 PRNP NPHP3 SEC14L1 RLBP1 NUDT19 TTPA USH2A ALDH3A2 ATXN3 LRAT TOPORS SYNJ1 MAK RP2 RBP1 ALPK3 FLVCR1 INVS CRX ATXN2 EFTUD2 PCDH15 CHM
191	9	PITPNM3 CNOT3 CDHR1 KIDINS220 NPTX2 SEMA6A TIMP2 MAPRE3 CERKL
26a	32	DYNLT3 COPS5 BBS1 RCC1 MVK SFRP2 PDE6A PLIN2 CEP290 LSM2 MYO7A IMPDH1 OTC SMC3 ABCG2 OTX2 PROM1 PRKCG RRH SOS2 SNRPB WDR19 AHI1 NRL PDC FGF18 PRPH KIZ FAM161A RAB8A NR2E3 RAB28
181b	26	FAM46A SOD3 GPR98 DHX38 MERTK TTC8 POC1B PRCD RPGRIP1 ADRA2B AGTPBP1 CDH23 STC1 NT5C2 TST EDNRA IMPG2 CC2D2A GUCA1B CXCR6 PRPF3 MT2A ARHGEF2 APOB PTPRC TUB

Supplementary Figure S1. Predicted target overlap for (a) *miR-183*, *miR-96*, and *miR-182* and (b) *miR-191*, *miR-26a*, and *miR-181b*. Target predictions were computed with CSMirTar using the human orthologues and restricting the output to genes associated with retinitis pigmentosa. Intersections were calculated using a Venn Diagram generator, available at http://bioinformatics.psb.ugent.be/webtools/Venn/

a

	mir-183	*mir-96*	*mir-182*
**WT**	CAGACTCCTGTTCTGTG**TATGGCA**CTGGTAGAATTCACTGTGAAA	GCGCTCTTCTTTGCCTGT**TTTGGCA**CTAGCACATTTTTGCTTTTTT	CAAAAGGTTCTCTGATGGTAT**TTGGCA**ATGGTAGAACTCACACTGGTG
lri60	CAGACTCCTGTTCTGTG**TATGGCA**CTGGTAGAATTCACTGTGAAA	GCGCTCTTCTTTGCC(Δ2)TT**TTGGCA**CTAGCACATTTTTGCTTTTTT CGCT	CAAAAGGTTCTCTGATGGTAT**TTGGCA**ATGGTAGAACTCACACTGGTG
lri61	CAGACTCCTGTTCTGTG**TATGGCA**CTGGTAGAATTCACTGTGAAA	GCGCTCTTCTTTGCCTGT**TTTGGCA**CTAGCACATTTTTGCTTTTTT	CAAAA(Δ11)GGTAT**TTGGCA**ATGGTAGAACTCACACTGGTG
lri69	--- DELETED ---	--- DELETED ---	CAAAAGGTTCTCTGATGGTAT**TTGGCA**ATGGTAGAACTCACACTGGTG
lri70	CAGACTT(Δ15)**CA**CTGGTAGAATTCACTGTGAAA	GCGCTCTTCTTTGCCTGT**TTTGGCA**CTAGCACATTTTTGCTTTTTT	CAAAAGGTTCTCTGATGGTAT**TTGGCA**ATGGTAGAACTCACACTGGTG
lri78	--- DELETED ---	--- DELETED ---	CAAAAGGTTCT(Δ1)TGATGGTAT**TTGGCA**ATGGTAGAACTCACACTGGTG TTATCTAATAGGTCTAAAGGTTTTTTATCTAATATCTAAA
lri79	CAGACTCCTGTTCTGTG**TATGGCA**CTGGTAGAATTCACTGTGAAA	GCGCTCTTCTTTGCC(Δ2)TT**TTGGCA**CTAGCACATTTTTGCTTTTTT CGCT	CAAAAGGTT(Δ4)GATGGTAT**TTGGCA**ATGGTAGAACTCACACTGGTG GAGCACATTATGAGG
lri81	CAGACTT(Δ15)**CA**CTGGTAGAATTCACTGTGAAA	GCGCTCTTCTTTGCCTGT**TTTGGCA**CTAGCACATTTTTGCTTTTTT	CAAAAGGTT(Δ3)TGATGGTAT**TTGGCA**ATGGTAGAACTCACACTGGTG GGTATTTGGTATTA

b

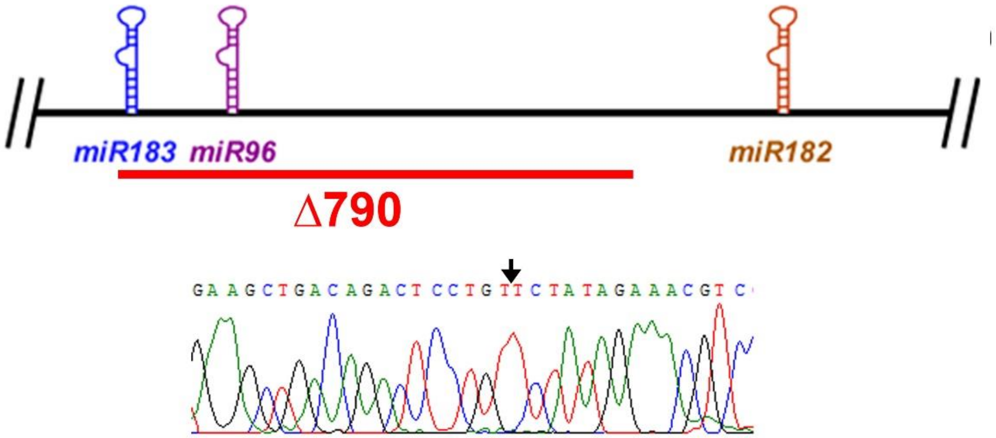

Supplementary Figure S2: (a) Sequence of miRNAs in wild-type and mutant alleles. The sequence of the mature miRNA is underlined, and the 6-mer minimal seed sequence is in bold. Alterations from the wild-type alleles are indicated in red. Allele designations are listed in the left column with the prefix "*lri*." Because these miRNAs are so tightly linked, they are inherited as a unit. We therefore consider compound mutations as unique alleles, even though they may have components in common with others. (b) The *lri69* and *lri78* mutations include a large, 790 base-pair deletion (red bar) that eliminates all portions of *mir-183* and *mir-96,* as well as the intervening sequence and a large amount of the region between *mir-96* and *mir-182*. Chromatogram shows the sequence of the mutation, with the arrow indicating the precise location of the deletion.

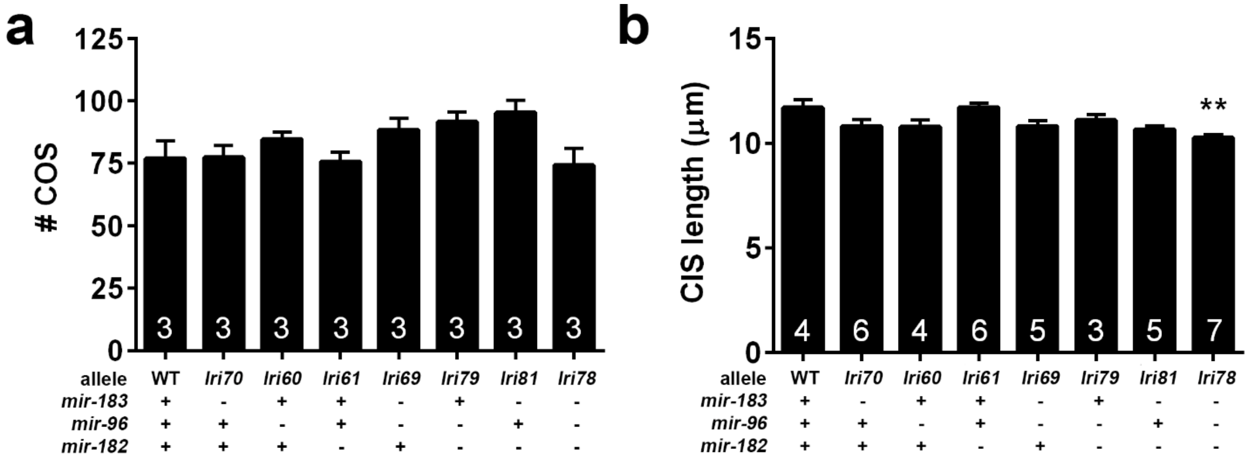

Supplementary Figure S3: Analysis of retinal structure in 5dpf mutants. (a) Cones were counted from retinal sections stained with PNA. None of the mutants are significantly different from controls (p=0.053, ANOVA). (b) Cone inner segment length was measured in retinal sections stained with zpr1. ** p=0.002, ANOVA with Dunnet's multiple comparisons test. COS = cone outer segment. CIS = cone inner segment. Numbers on bars indicate sample size.

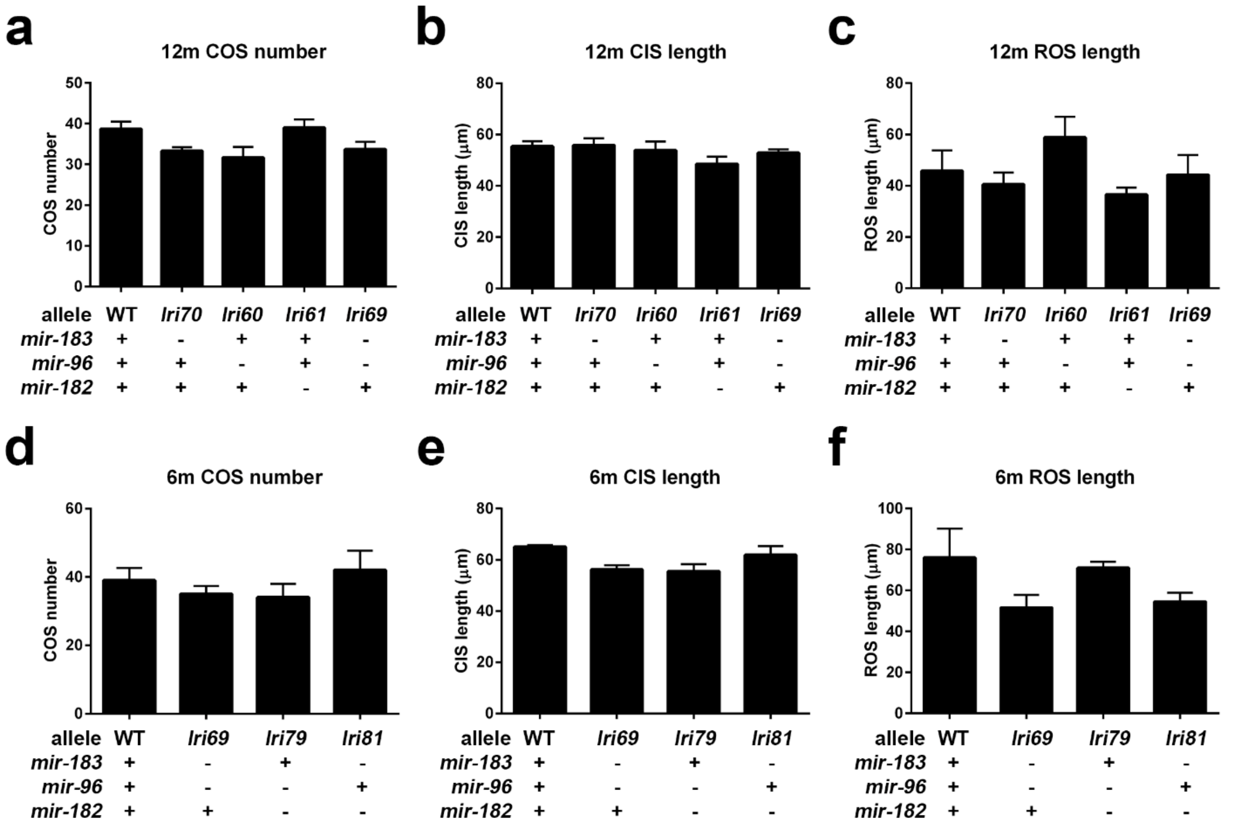

Supplementary Figure S4: Retinal structure was analyzed in adult fish at either (a-c) 12 months or (d-f) 6 months. (a, d) Cones were counted from retinal sections stained with PNA. (b, e) Cone inner segment length was measured from sections stained with zpr1. (c,f) Rod outer segment length was measured from sections stained with zpr3. In each case, mutants were not significantly different from controls (ANOVA). COS = cone outer segment. CIS = cone inner segment. ROS = Rod outer segment. (a) p = 0.074. (b) p = 0.337. (c) p = 0.239. (d) p = 0.551. (e) p = 0.073. (f) p = 0.226. For all data points, n=3.

Supplementary Table S1: Oligonucleotides used for gene targeting, cloning, and genotyping

**Gene targeting oligos, hybridized and cloned into pDR274**	
mir-183 crisprF	TAGGAGACTCCTGTTCTGTGTA
mir-183 crisprR	AAACTACACAGAACAGGAGTCT
mir-96 crisprF	TAGGATGTGCTAGTGCCAAAAC
mir-96 crisprR	AAACGTTTTGGCACTAGCACAT
mir-182 crisprF	TAGGGGACAAAAGGTTCTCTGA
mir-182 crisprR	AAACTCAGAGAACCTTTTGTCC
**Genotyping primers, used for either HRMA or agarose gel electrophoresis**	
mir-183HRMF	GCCGGAGTCTTAAAGGGTGA
mir-183HRMR	GGCCCTTTGGTAATTCACTGA
mir-96HRMF	ACCACGTGTTTGATTTGCTCTC
mir-96HRMR	TGTCCCATATTGGCACTACACA
mir-182HRMF	GAATGGCCCATTTCTTCCCC
mir-182HRMR	CCGGATCTGACTACCTCACC
**Primers for cluster sequencing, and for genotyping the mir-183/96^{lri69} allele**	
183cSeqF	TACGCTCCCCCATTCCCTAC
183cSeqR	ATTTCAGCCCAACCTGCACT
**Oligos for insertion of SanDI site into p3E-pA**	
pA+SanDI-F	CTTGTACAAAGTGGGGGGTCCCGGATCCAGACATGAT
pA+SanDI-R	ATCATGTCTGGATCCGGGACCCCCCACTTTGTACAAG
**Oligos for inserting miRNA binding sites into p3E-SanDI**	
miR183MBS-AS	5'P-GACCCTATGGCACTGGTAGAATTCACTGAATTTATGGCACTGGTAGAATTCACTGGG
miR183MBS-S	5'P-GTCCCCAGTGAATTCTACCAGTGCCATAAATTCAGTGAATTCTACCAGTGCCATAGG
miR96MBS-AS	5'P-GACCCTTTGGCACTAGCACATTTTTGCTAATTTTTGGCACTAGCACATTTTTGCTGG
miR96MBS-S	5'P-GTCCCAGCAAAAATGTGCTAGTGCCAAAAATTAGCAAAAATGTGCTAGTGCCAAAGG
miR182MBS-AS	5'P-GACCCTTTGGCAATGGTAGAACTCACAAATTTTTGGCAATGGTAGAACTCACAGG
miR182MBS-S	5'P-GTCCCTGTGAGTTCTACCATTGCCAAAAATTTGTGAGTTCTACCATTGCCAAAGG
miR129MBS-AS	5'P-GACCCAAGCCCTTACCCCAAAAAGCATAATTAAGCCCTTACCCCAAAAAGCATGG
miR129MBS-S	5'P-GTCCCATGCTTTTTGGGGTAAGGGCTTAATTATGCTTTTTGGGGTAAGGGCTTGG
**For cloning miRNA loci**	
mir183cloneF	ATGCCGGCCGTACGCTCCCCCATTCCCTAC
mir183cloneR	TACGCCTGAGGTGTGCTAGTGCCAAAACAGG
mir96cloneF	ACATCGGCCGGGGCCATAAACAGAGCAGAGA
mir96cloneR	TACGCCTGAGGTTCCGCAGTAAAGGTCGCTT
miR182cloneF	ATGCCGGCCGAGTTTAGTATCATTCAGTATGTCCGA
miR182cloneR	TGCACCTGAGGTCATTGAAACAACCTTGATATTCGA
miR129cloneF	ATGCCGGCCGCGGGTGCCTACGTCATCTAC
miR129cloneR	ATGCCCTGAGGTGCAGAAAACTGAATTGAAAGGCT

Supplementary Video S1: Swimming behavior of 15 week old *mir-183/96/182*^{lri78} mutants. Only the two small fish with erratic swimming behavior are homozogytes.

Supplementary Video S2: Swimming behavior of 17 month old *mir-183/96*^{lri69} mutants. All of these fish are homozygotes, but exhibit variable penetrance of the phenotype.
